# The Anti-Tumor Effect of Boron Neutron Capture Therapy in Glioblastoma Subcutaneous Xenograft Model Using the Proton Linear Accelerator-Based BNCT System in Korea

**DOI:** 10.3390/life12081264

**Published:** 2022-08-19

**Authors:** Il Hyeok Seo, Jeongwoo Lee, Dasom Na, Hyunhye Kyung, Jieun Yang, Sangbong Lee, Sang June Jeon, Jae Won Choi, Kyu Young Lee, Jungyu Yi, Jaehwan Han, Mooyoung Yoo, Se Hyun Kim

**Affiliations:** 1 A-BNCT Center, Dawonmedax, Incheon 21988, Korea; 2 Department of Pharmacy, ERICA Campus, Hanyang University, Ansan 15588, Korea; 3 Department of Nuclear Engineering, Hanyang University, Seoul 04763, Korea

**Keywords:** BNCT, BPA, linear accelerator, radiation therapy, Korea

## Abstract

Boron neutron capture therapy (BNCT) is a radiation therapy that selectively kills cancer cells and is being actively researched and developed around the world. In Korea, development of the proton linear accelerator-based BNCT system has completed development, and its anti-cancer effect in the U-87 MG subcutaneous xenograft model has been evaluated. To evaluate the efficacy of BNCT, we measured ^10^B-enriched boronophenylalanine (BPA) uptake in U-87 MG, FaDu, and SAS cells and evaluated cell viability by clonogenic assays. In addition, the boron concentration in the tumor, blood, and skin on the U-87 MG xenograft model was measured, and the tumor volume was measured for 4 weeks after BNCT. In vitro, the intracellular boron concentration was highest in the order of SAS, FaDu, and U-87 MG, and cell survival fractions decreased depending on the BPA treatment concentration and neutron irradiation dose. In vivo, the tumor volume was significantly decreased in the BNCT group compared to the control group. This study confirmed the anti-cancer effect of BNCT in the U-87 MG subcutaneous xenograft model. It is expected that the proton linear accelerator-based BNCT system developed in Korea will be a new option for radiation therapy for cancer treatment.

## 1. Introduction

Boron neutron capture therapy (BNCT) is a radiation therapy that selectively kills cancer cells [1]. The principle of BNCT is that a nuclear reaction occurs when boron-10 (^10^B) accumulates in cancer cells and captures thermal neutrons, and the DNA double-strand of the cancer cell is destroyed by the generated alpha particles and lithium ions [2,3]. These particles are known to have an energy length range of 5–9 μm in the tissue [4]. BNCT was first proposed by Locher in 1936 for use in the treatment of cancer tumors [5] and Sweet in 1951 for use in the treatment of most malignant brain tumors [6]. Soon, clinical trials were conducted at the Massachusetts Institute of Technology (MIT) using neutrons from a research reactor and non-selective boron drug, but BNCT was stopped in 1961 due to potential side effects and toxicity [2,7]. Since tumor selective boron compounds were developed, clinical results have shown therapeutic efficacy, associated with an improvement in patient quality of life and prolonged survival. BNCT was studied for the treatment of several pathologies and has been performed or is underway in the United States, Japan, European Consortium, Sweden, Italy, Finland, Argentina, and Taiwan (among others), employing mostly nuclear reactors as the neutron source [8,9,10,11,12,13]. Recently, accelerator-based neutron generators are undergoing development worldwide because accelerator-based generators have fewer radiation hazards and are suitable for installation in hospitals compared to reactor-based generators [14,15]. In Japan, a cyclotron-type, accelerator-based neutron generator has been developed and approved to treat patients with head and neck cancer [16,17]. In Korea, a linear accelerator-based neutron generator has been developed (DM-BNCT; Dawonmedax, Seoul, Korea). DM-BNCT (10 MeV, 4 mA) is a linear accelerator that generates neutrons using a beryllium target after accelerating protons using radio-frequency-quadruple (RFQ) and drift tube LINAC (DTL).

As part of the first generation of boron drugs, boric acid was used in clinical trials in the 1950s and 1960s but had low tumor selectivity [17]. Sodium borocaptate (BSH) and boronophenylalanine (BPA) were developed as the second generation [17,18]. BSH and BPA are still used as boron drugs for accumulating ^10^B in cancer cells [18]. BSH is a compound containing 12 boron atoms and has high potential as a boron drug, but its targeting of cancer cells is limited [19]. Therefore, new drugs targeting cancer cells, including BSH compounds, are being studied [20,21]. BPA is an amino-acid derivative and one of the essential amino acids, which accumulates into cells through L-type amino acid transporter (LAT1), and LAT1 is overexpressed in cancer cells [22,23]. Therefore, BPA is widely used in BNCT clinical trials to treat cancer patients [3].

In this study, BNCT was performed using BPA as a boron drug and a linear accelerator-based neutron generator (DM-BNCT). To evaluate the efficacy of BNCT, the anti-cancer effect was investigated on a glioblastoma (GBM) cell line subcutaneous xenograft model.

## 2. Materials and Methods

### 2.1. Cell Culture

The human glioblastoma cell line U-87 MG and human pharynx squamous carcinoma cell line FaDu were obtained from the Korean cell line bank (KCLB; Seoul, Korea). The human tongue squamous carcinoma cell line SAS was obtained from the Japanese collection of research bioresources cell bank (JCRB; Tokyo, Japan). U-87 MG and FaDu cells were cultured in minimum essential medium (MEM) (Gibco, New York, NY, USA) with 2 mM L-glutamine (Gibco), 100 units/mL penicillin-streptomycin (Gibco), and 10% (*v/v*) fetal bovine serum (Gibco) at 37 °C under a 5% CO_2_ atmosphere. SAS cells were cultured in Dulbecco’s modified eagle medium/Ham’s F-12 (DMEM/F12) (Gibco) with 100 units/mL penicillin-streptomycin (Gibco) and 10% (*v/v*) fetal bovine serum (Gibco) at 37 °C under a 5% CO_2_ atmosphere.

### 2.2. Boron Compounds

^10^B-enriched (>99%) boronophenylalanine (BPA) was obtained from Interpharma Praha (Praha, Czech) and dissolved with D-sorbitol (Roqeutte, Lestrem, France). BPA and D-sorbitol were mixed at a ratio of 1:1.05 and then dissolved with NaOH (Samchun, Seoul, Korea). The pH was adjusted with HCl (Duksan, Ansan, Korea). The concentration of BPA was adjusted to 10% (*w/v*) with sterilized distilled water (DW). The final concentration of BPA was 100 mg/mL. Finally, it was filtered using a 0.22 μm filter.

### 2.3. In Vitro Uptake of Boron

U-87 MG, FaDu, and SAS cells were seeded 5 × 10^5^ cells/well into a 6-well cell culture plate (SPL, Pocheon, Korea) with culture medium at 37 °C with 5% CO_2_ for 24 h. Cells were treated with 500, 1000, and 2000 μg/mL (≒24, 48, and 96 μg [^10^B]/mL) of BPA for 3 h. The medium was removed, and cells were harvested into 1.5 mL tube using Trypsin-EDTA (Gibco). Cells were centrifuged and the supernatant discarded. Intracellular boron concentration was measured by inductively coupled plasma mass spectrometry (ICP-MS). 

### 2.4. BNCT In Vitro Efficacy Experiment: Clonogenic Assay

**Figure 1:**
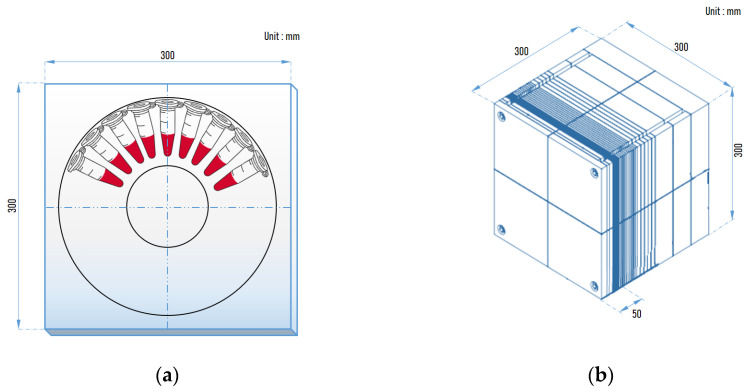
Preparation of in vitro neutron irradiation. (**a**) The 1.5 mL tubes containing the cells were attached to a plate of an acrylic phantom; (**b**) acrylic phantom for neutron irradiation; the cells were located about 5 cm in front of the acrylic phantom.

U-87 MG, FaDu, and SAS cells were seeded 5 × 10^5^ cells/well into 6-well cell culture plate with culture medium at 37 °C with 5% CO_2_ for 24 h. Cells were treated with 500 or 1000 μg/mL of BPA for 3 h. The medium was removed, and cells were harvested into 1.5 mL tubes using Trypsin-EDTA. Cells were centrifuged, and the supernatant discarded. The BPA containing medium was placed in a 1.5 mL tube, and the cells were resuspended. For neutron irradiation, tubes containing U-87 MG, FaDu, or SAS cells were attached to a plate of an acrylic phantom (Figure 1) and irradiated with a thermal neutron fluence of 2, 3, and 4 × 10^11^ n/cm^2^, respectively. Neutron irradiation was conducted by DM-BNCT. Details about the accelerator and beam parameters can be found in Lee et al. as well as an overview of the A-BNCT system in Korea [24,25]. To accurately measure the thermal neutron fluence, we used two methods: one was the foil activation method, and the other was the Eu:LiCAF scintillation detector. The foil activation method is well-known for neutron flux measurements; we used Au foil and Cd-covered Au foil to measure the thermal neutron fluence [26], and the Eu:LiCAF scintillator is mainly sensitive to thermal neutrons and has low sensitivity to the epithermal and fast neutron energy ranges [27,28]. After neutron irradiation, the control group and neutron irradiation control group were seeded with 200 cells, the U-87 MG BNCT group was seeded with 400 cells, and for the FaDu and SAS BNCT groups, 1600 cells were seeded in the 60 mm cell culture dish (SPL) and incubated at 37 °C with 5% CO_2_ for up to 8 days for SAS, 10 days for FaDu, and 11 days for U-87 MG cells. The appropriate number of seeds and incubation time of U-87 MG, FaDu, and SAS were set through preliminary tests. The appropriate number of colonies for clonogenic assay is 20–150 [29]. The cells were washed twice with DPBS, fixed in 10% neutral-buffered formalin solution (Sigma-Aldrich, St. Louis, MO, USA) for 30 min, and stained with 0.01% crystal violet (Sigma-Aldrich) for 60 min. Colonies of more than 50 cells were counted [29,30].

### 2.5. Biodistribution In Vivo Experiment

All procedures were performed in accordance with protocols approved by the Institutional animal care and use committee of Korea Institute of Radiological Medical Sciences (KIRAMS; No. kirams2020-0007). Certified rodent diet (Envigo, Indianapolis, IN, USA) and water were supplied ad libitum. The room temperature was about 22 ± 3 °C with a 12/12 h light/dark cycle. Cage changing was performed once a week. After all mice were acclimatized for 1 week, 5 × 10^6^ U-87 MG human glioblastoma cells were subcutaneously injected in the left thigh of 7-week-old BALB/c-nu/nu mice. When tumors reached a target volume of approximately 100 mm^3^, all mice were intravenously administered with BPA 500 and 1000 mg/kg. Mice were anesthetized using 2~3% isoflurane induction and 1.5~2.5% for maintenance with 99.5% oxygen. Blood, skin, and tumors were collected at 0.17, 0.5, 1, 2, 3, and 5 h after injection. Blood samples were taken by cardiac puncture prior to mouse sacrifice and collected in blood collection tubes. After all mice were sacrificed, tumor and skin samples were obtained and stored in 15 mL conical tubes. All samples were stored in a −80 °C deep freezer until boron (^10^B) concentration measurements.

### 2.6. ICP-MS Analysis

The cells were digested with 2% nitric acid solution (Sigma-Aldrich) for 20 min and centrifuged. The cell debris was discarded, and internal standard (yttrium) was added. The boron (^10^B) concentration in the supernatant was measured using ICP-MS (PerkinElmer NexION 2000B, PerkinElmer Inc., Waltham, MA, USA) with high-purity argon. The blood, skin, and tumor were digested with 70% nitric acid solution at 100 °C for 1 h in Thermomixer (Eppendorf, Hamburg, Germany) and dilution with water and internal standard (yttrium). The boron (^10^B) concentration was measured using ICP-MS; (PerkinElmer NexION 300D, PerkinElmer Inc., USA) with high-purity argon. Confirmation of the reliability of the measurement method was confirmed using six concentrations prepared using the standard solution.

### 2.7. BNCT In Vivo Efficacy Experiment

**Figure 2:**
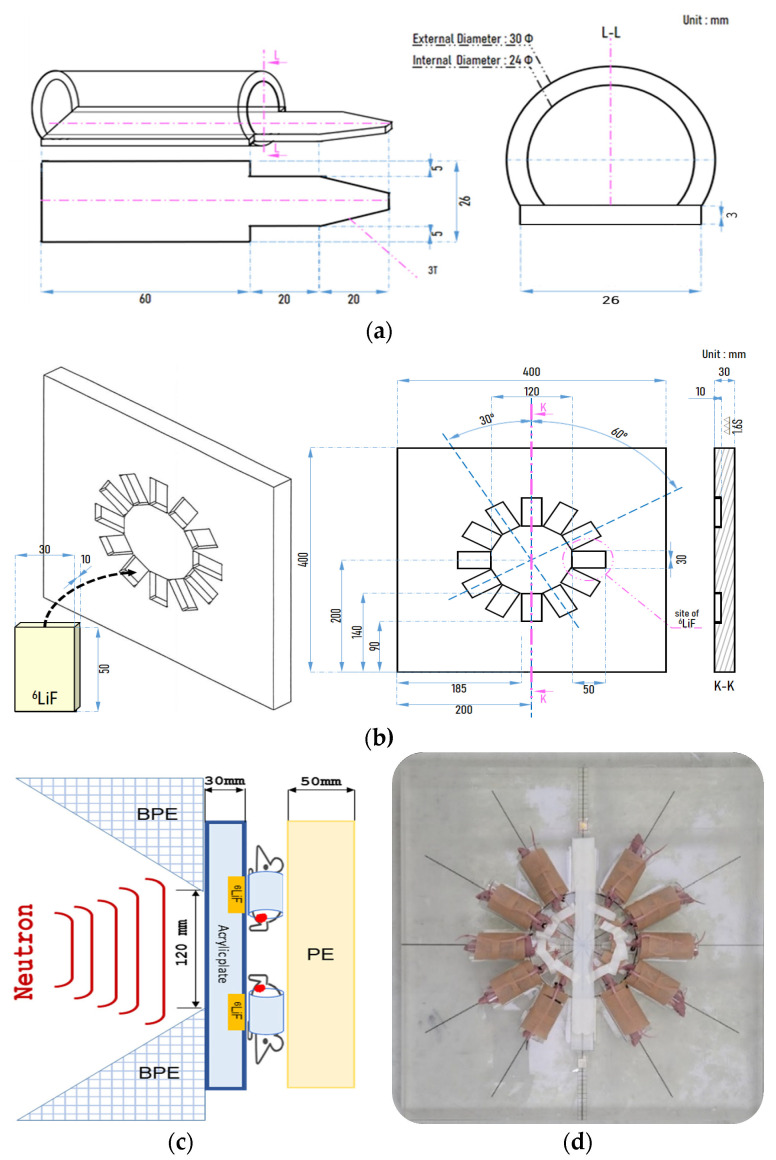
Preparation of in vivo neutron irradiation. For neutron irradiation of mice, fixation is required to prevent them from moving around. (**a**) Acrylic cylinder for fixing the mouse; (**b**) acrylic and ^6^LiF plate for neutron irradiation of mice; (**c**,**d**) image of mice fixed on the acrylic cylinder and plate under irradiation with neutrons. BPE, borated polyethylene; PE, polyethylene.

All procedures were performed in accordance with protocols approved by the Institutional animal care and use committee of Croen Inc. (Suwon, Korea; No. 21M024). Certified rodent diet (Envigo) and water were supplied ad libitum. The room temperature was about 22 ± 3 °C with a 12/12 h light/dark cycle. Cage changing was performed once a week. To observe the decrease in tumor volume in the U-87 MG animal model, after all mice were acclimatized for 1 week, 1 × 10^6^ U-87 MG human glioblastoma cells were subcutaneously injected in the left thigh of 7-week-old female BALB/c-nu/nu mice. The day of injection of U-87 MG cells was defined as day 1 and day 7 from the date of injection, which was counted as 1 week. When the tumor volume grew to 100 ± 20 mm^3^, mice were divided into 5 groups: Group 1 was an untreated control group that was not exposed to radiation or BPA. Group 2 was the neutron irradiation group without BPA administration (irradiation only). Groups 3, 4, and 5 were BNCT groups that were irradiated with skin doses of 4, 5, and 6 Gy-Eq, after BPA injection, (groups 3, 4 are 500 mg/kg injection, and group 5 is 1000 mg/kg injection), respectively. Mice were transferred to the A-BNCT center (Dawonmedax, Korea), and BPA 500 and 1000 mg/kg were administered intravenously 1 h before neutron irradiation. Each mouse was fixed in a specially designed acrylic cylinder for neutron irradiation and then placed on acrylic (30 mm thick) and ^6^LiF (10 mm thick) plates to shield the body from thermal neutrons while the tumor-inoculated left thigh was exposed. (Figure 2). Mice were irradiated with neutron at doses corresponding to skin doses 4, 5, and 6 Gy-Eq by DM-BNCT. Tumor and skin doses by BNCT were calculated by the treatment-planning system (DM-BTPS; Dawonmedax). Dose calculation was performed using the average boron concentration from 1 h to 2 h, analyzed by ICP-MS in biodistribution during an in vivo experiment. The absorbed dose of BNCT (Gy-Eq) is calculated by four physical doses, which are boron dose (D_B,ppm_), nitrogen dose (D_N_), hydrogen dose (D_H_), and gamma dose (D_γ_) [23]. Compound biological effectiveness (CBE) and relative biological effectiveness (RBE) were considered [31]. The formula is: Gy-Eq = CBE_B_ × D_B,ppm_ + RBE_N_ × D_N_ + RBE_H_ × D_H_ + RBE_γ_ × D_γ_ [23]. The calculated absorbed doses to tumor and skin by BNCT are listed in Table 1. Tumor volume measurements were performed three times a week for 4 weeks using a Vernier caliper, and tumor volume was calculated using the following formula: tumor volume (mm^3^) = (long axis × short axis^2^)/2.

## 3. Results

### 3.1. In Vitro Uptake of Boron (10B)

To measure the intracellular boron concentration, U-87 MG, FaDu, and SAS cells were treated with BPA at 500, 1000, and 2000 μg/mL for 3 h, respectively. Intracellular boron (^10^B) concentrations were measured by ICP-MS. The intracellular boron concentrations in U-87 MG were 3.9 ± 0.1, 7.0 ± 0.4, and 11.1 ± 0.8 ng/10^5^ cells; those in FaDu were 5.9 ± 0.4, 12.8 ± 1.1, and 23.6 ± 1.7 ng/10^5^ cells; and those in SAS were 10.8 ± 1.8, 19.1 ± 0.5, and 36.6 ± 1.2 ng/10^5^ cells (Table 2). It was confirmed that the intracellular boron concentration increased in U-87 MG, FaDu, and SAS depending on the BPA treatment concentration.

### 3.2. BNCT In Vitro Efficacy Experiment

**Figure 3:**
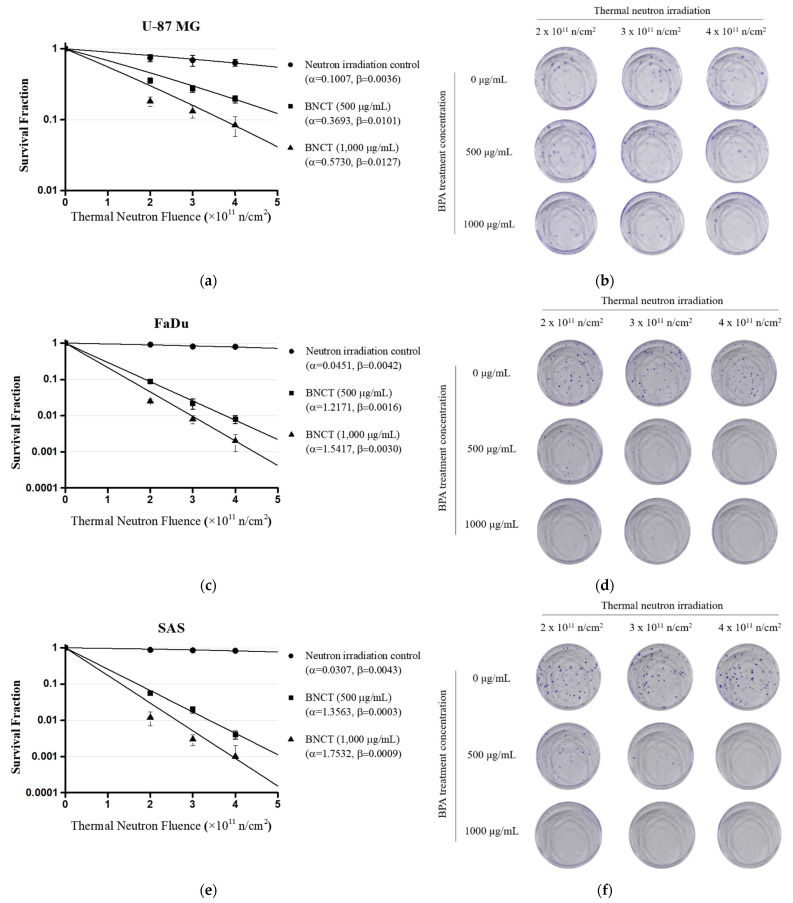
Cell viability estimation after BNCT. U-87 MG, FaDu, and SAS cells were irradiated with thermal neutrons at 2, 3, and 4 × 10^11^ n/cm^2^ after treatment with BPA at 500 and 1000 μg/mL for 3 h. (**a**) U-87 MG cell survival fraction after BNCT; (**b**) U-87 MG cell colonies according to BPA treatment concentration and thermal neutron dose; (**c**) FaDu cell survival fraction after BNCT; (**d**) FaDu cell colonies according to BPA treatment concentration and thermal neutron dose; (**e**) SAS cell survival fraction after BNCT; (**f**) SAS cell colonies according to BPA treatment concentration and thermal neutron dose. Data are represented as the mean ± SD (n = 3). Radiobiological parameters (α = alpha, β = beta), n, number of plates.

To evaluate the in vitro efficacy of BNCT mediated by BPA, U-87 MG, FaDu, and SAS cells were treated with BPA 500 and 1000 μg/mL for 3 h and exposed to thermal neutron irradiation (2, 3, and 4 × 10^11^ n/cm^2^). Cell viability was evaluated using clonogenic assay. The survival fractions of U-87 MG cells were 0.358, 0.274, and 0.196 in the 500 μg/mL treatment group and 0.182, 0.132, and 0.084 in the 1000 μg/mL treatment group (Figure 3a,b). The survival fractions of FaDu cells were 0.087, 0.022, and 0.008 in the 500 μg/mL treatment group and 0.025, 0.008, and 0.002 in the 1000 μg/mL treatment group (Figure 3c,d). The survival fractions of SAS cells were 0.056, 0.020, and 0.004 in the 500 μg/mL treatment group and 0.012, 0.003, and 0.001 in the 1000 μg/mL treatment group (Figure 3e,f). In the U-87 MG, FaDu, and SAS BNCT groups, it was confirmed that the survival fraction decreased depending on the BPA treatment concentration and neutron irradiation dose, respectively.

### 3.3. Biodistribution In Vivo Experiment

**Figure 4:**
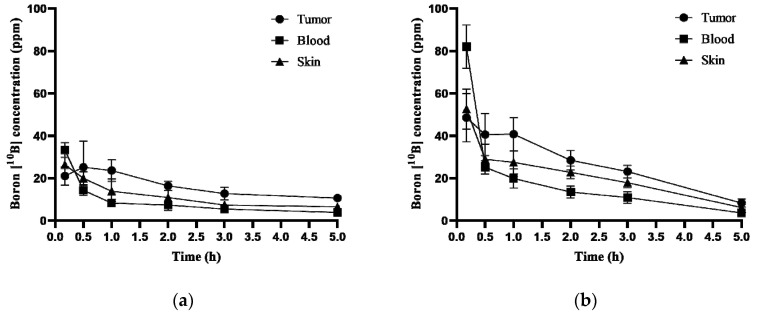
Biodistribution of BPA in the U-87 MG subcutaneous xenograft model. Measurement of boron concentration in tumor, blood, and skin at 0.17, 0.5, 1, 2, 3, and 5 h after administration of BPA 500 and 1000 mg/kg i.v. injection in U-87 MG subcutaneous xenograft model. (**a**) Boron concentration in tumor, blood, and skin after BPA 500 mg/kg administration; (**b**) boron concentration in tumor, blood, and skin after BPA 1000 mg/kg administration. BPA 500 mg/kg administration group, n = 4; BPA 1000 mg/kg administration group, n = 4 (5 h group, n = 3). Data are represented as the mean ± SD. n, number of animals.

To measure the boron concentration in the U-87 MG subcutaneous xenograft model, tumor, blood, and skin were collected at 0.17, 0.5, 1, 2, 3, and 5 h after being administered intravenously with BPA 500 and 1000 mg/kg. Boron (^10^B) concentrations in tumor, blood, and skin were measured by ICP-MS. In the 500 mg/kg injection group, the tumor boron concentrations were 21.1, 25.2, 23.7, 16.4, 12.8, and 10.7 ppm, and the blood boron concentrations were 33.3, 14.4, 8.3, 7.4, 5.5, and 3.9 ppm. In the 1000 mg/kg injection group, the tumor boron concentrations were 48.6, 40.6, 40.8, 28.5, 23.2, and 8.3, and the blood boron concentrations were 82.1, 25.2, 19.9, 13.5, 10.9, and 3.7 ppm (Figure 4). The boron (^10^B) biodistribution data, including tumor–blood boron concentration ratio (T/B ratio) in the U-87 MG subcutaneous xenograft model, are listed in Table 3.

### 3.4. BNCT In Vivo Efficacy Experiment

**Figure 5:**
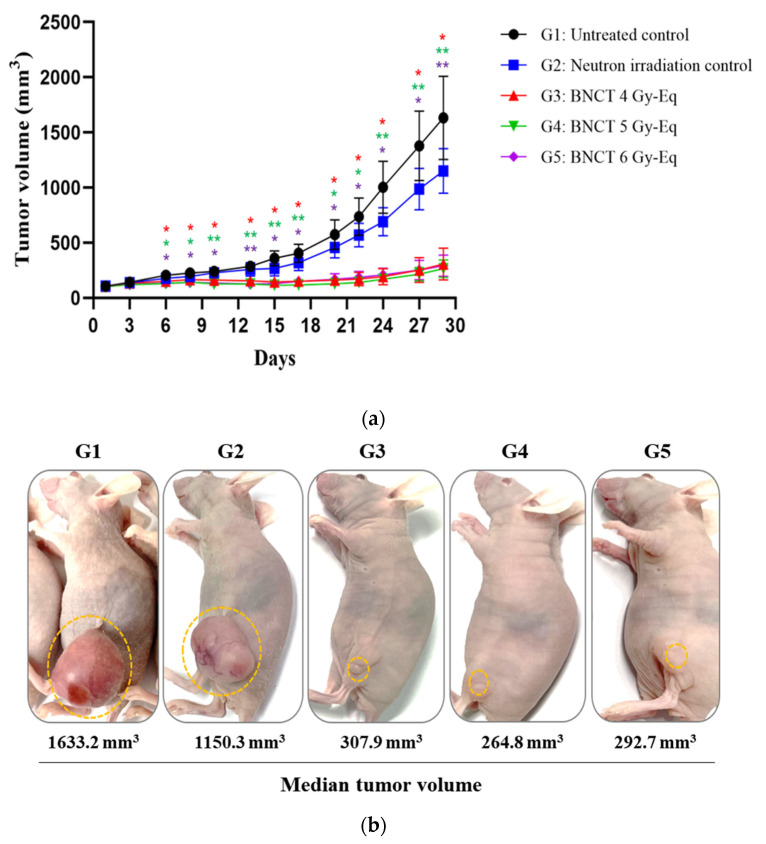
Efficacy of BNCT to inhibit tumor growth. After 1 h of administration of BPA 500 and 1000 mg/kg by i.v. injection to the U-87 MG subcutaneous xenograft model, neutron irradiation with skin doses of 4, 5, and 6 Gy-Eq was performed. (**a**) Tumor growth as determined by measurement of tumor volume for 4 weeks. (**b**) After 4 weeks of neutron irradiation, G1~G5 visual tumor size. G1, n = 8; G2~G5, n = 10. Data are represented as the mean ± SED. * *p* < 0.05, ** *p* < 0.01, *** *p* < 0.001. n, number of animals.

Tumor growth was measured by tumor volume in the untreated control group (G1) and all the irradiated groups (G2~G5) for 4 weeks from the irradiation day. The untreated control group (G1) and neutron irradiation group without BPA administration (G2) showed rapid tumor growth, and the average tumor volumes at 4 weeks after neutron irradiation were 1633.2 mm^3^ and 1150.3 mm^3^. However, the BNCT group with a skin dose of 4 Gy-Eq (G3) showed a significant decrease in tumor volume from the 13th day after irradiation, and the tumor volume was 307.9 mm^3^ at 4 weeks after irradiation (* *p* < 0.05). The BNCT group with skin doses of 5 and 6 Gy-Eq (G4, G5) showed a significant decrease in tumor volume from the 6th and 8th days, respectively, compared to the untreated control group (G1), and the tumor volume was 264.8 and 292.7 mm^3^ at 4 weeks after irradiation (** *p* < 0.01). All the *p*-values were calculated by ANOVA (Figure 5a). Under visual observation, almost no tumor was detected in G3, G4, and G5 compared to G1 and G2 (Figure 5b).

## 4. Discussion

To evaluate BNCT therapeutic efficacy in vitro and in vivo at the proton linear accelerator-based neutron generator (DM-BNCT), we needed to establish factors such as BPA treatment concentration and time, boron concentration in cells and tumor, and neutron irradiation time point. To establish BNCT in vitro efficacy experiment conditions, we measured the intracellular boron concentration by treating 500, 1000, and 2000 μg/mL of BPA in cells for 3 h. BPA is effluxed in cells within a short time in a medium without BPA [32]. For this reason, neutron irradiation is carried out in the presence of BPA in the medium [33,34,35]. Therefore, in order to maintain the intracellular BPA concentration, we used a medium containing BPA. The intracellular boron concentration with BNCT efficacy was about 41.4 ± 2.5 ng/10^6^ cells, which was confirmed by Kanemitsu et al. [36]. Therefore, we established BPA treatment concentration and time conditions in U-87 MG cells at 1000 μg/mL for 3 h. To establish BNCT in vivo efficacy experiment conditions, we considered the boron concentration and tumor–blood boron concentration ratio (T/B ratio) [37,38]. The boron concentration at 0.17, 0.5, 1, 2, 3, and 5 h after BPA 500 and 1000 mg/kg administration was measured in the U-87 MG subcutaneous xenograft model, and it was confirmed that the boron concentration was high within the tumor, and the duration for which the high T/B ratio was maintained was between 1 and 2 h after BPA administration. Therefore, we established a condition in which neutron irradiation proceeds from 1 to 2 h after BPA administration. Based on these established conditions, we conducted BNCT in vitro and in vivo efficacy experiments. As a result, it was suggested that GBM cell line U-87 MG and HNC cell line FaDu and SAS have BNCT-induced cell death and that there is anti-cancer effect of BNCT in the U-87 MG subcutaneous xenograft model.

One of the most important factors of BNCT is boron drugs. The boron drugs should deliver intracellular boron specifically to cancer cells. A representative boron drug is BPA, which passes through the LAT1 transporter that is overexpressed in cancer cells and accumulates intracellular boron specifically in cancer cells [39]. In addition, BPA has been reported to penetrate the blood–brain barrier (BBB) [40,41]. We treated BPA to U-87 MG and FaDu and SAS to measure intracellular boron concentration to evaluate BPA uptake in cancer cells, and the results were the highest in the order of SAS, FaDu, and U-87 MG. According to Yoshimoto (2018), the expression of LAT1 was higher in FaDu than in U-87 MG [42], and it was confirmed that the higher the LAT1 expression, the higher the uptake of BPA. This means that the higher the BPA uptake, the higher the anti-cancer effect of BNCT. In addition, if there is an anti-cancer effect of BNCT in the U-87 MG subcutaneous xenograft model, which is the lowest BPA uptake, we could suggest that a better anti-cancer effect of BNCT could be reported in other cells with higher BPA uptake (FaDu, SAS). However, radiobiological studies are needed to confirm this statement. Therefore, U-87 MG, which had a low intracellular boron concentration, was subcutaneously injected in nude mice (glioblastoma xenograft model), and after being intravenously administered with BPA, the boron concentration in the tumor was measured to confirm that BPA was uptaken into the tumor. It was confirmed that BPA was suitable for our BNCT system as a boron drug. 

Another important BNCT factor is neutrons. Accelerator-based neutron generator types include electrostatic, cyclotron, and linear [43]. In Korea, a linear accelerator-based neutron generator has been developed. Although the linear accelerator (RFQ + DTL)-based neutron generator is difficult to manufacture, the residual radiation is low [44]. The therapeutic principle of BNCT is that alpha particles and lithium ions generate DNA double-strand breaks in cancer cells that induce cell death. These particles are generated when the boron accumulated in cancer cells captures a thermal neutron [3,45]. In the case of deep-seated tumors, to deliver thermal neutrons into cancer cells at a certain depth, the neutron accelerator generates epithermal neutrons. Epithermal neutrons pass through tissues and are decelerated to thermal neutrons at the depths of cancer cells [4]. In the case of cells or mice, acrylic (polymethyl methacrylate; PMMA) material, which has similar properties to human tissue, is mainly used for the thermalization of epithermal neutrons [46,47]. In this study, the acrylic cylinder, acrylic plate (in vivo), and acrylic phantom (in vitro) were designed and manufactured so that sufficient thermal neutrons could reach cancer cells and mouse tumors and were applied to the in vitro and in vivo efficacy experiments of BNCT.

There are several limitations although we conducted in vitro and in vivo experiments using the proton linear accelerator-based BNCT system in this study. First, we performed in vitro uptake of boron and BNCT in vitro efficacy experiments in glioblastoma and head and neck cancer cells, but experiments should be performed on more types of cancer cells to further expand cancer treatment. Second, only the glioblastoma subcutaneous xenograft model was used in the in vivo BNCT efficacy experiment, but if BNCT research is conducted in various animal models, including orthotopic models, in the future, more efficient clinical trials and therapeutic effects are expected. In addition, the neutron control survival fraction in the BNCT in vitro efficacy experiment is higher than the neutron control survival fraction in other BNCT experiments performed by other groups. We consider this to be due to the beam character of the reactor or accelerator of each BNCT experiment [33,48,49,50]. However, comparison of experimental results between BNCT groups is limited due to cells, tumor size at the time of irradiation, BPA concentration, neutron irradiation, and conditions. Lastly, we focused on BNCT in vitro and in vivo experiments in this manuscript and so only briefly mentioned the BNCT dosimetry and treatment-planning system. The BNCT dosimetry is complicated because different beam components are mixed, and each has different RBE [51]. Therefore, research on BNCT dosimetry is important and essential and will be addressed in the future.

We are preparing a clinical trial for the treatment of cancer patients using the proton linear accelerator-based BNCT system. The established conditions used to evaluate the BNCT efficacy in vitro and in vivo could provide important data for BNCT clinical trials and approval.

## 5. Conclusions

In this study, neutron irradiation was performed with a developed proton linear accelerator-based neutron generator (DM-BNCT). It was confirmed that the survival fraction decreased depending on the BPA treatment concentration and neutron irradiation dose in U-87 MG, FaDu, and SAS cells. In addition, we confirmed the anti-cancer effect by observing the decrease in tumor volume in the U-87 MG subcutaneous xenograft model using BNCT. The proton linear-accelerator-based BNCT system developed in Korea is a very promising cancer treatment for glioblastoma patients for whom tumor treatment has traditionally been difficult. In the future, it is expected that boron neutron capture therapy will be a milestone in the field of radiation therapy for cancer treatment.

## Figures and Tables

**Table 1:** Absorbed dose of skin and tumor by BNCT.

Group	Absorbed Dose (Gy-Eq)	
	Skin	Tumor
G3	4.0	9.8
G4	5.0	12.2
G5	6.0	17.9

Dose calculations for skin and tumor were performed with DM-BTPS. When the skin dose by BNCT was 4.0, 5.0, and 6.0, the tumor dose was calculated as 9.8, 12.2, and 17. 9 Gy-Eq, respectively.

**Table 2:** Intracellular boron (^10^B) concentration in U-87 MG, FaDu, and SAS cells.

Cell	BPA Treatment Concentration (μg/mL)	Intracellular Boron Concentration (ng/10^5^ Cells)
U-87 MG	500 (≒24 μg [10B]/mL)	3.9 ± 0.1
	1000 (≒48 μg [10B]/mL)	7.0 ± 0.4
	2000 (≒96 μg [10B]/mL)	11.1 ± 0.8
FaDu	500 (≒24 μg [10B]/mL)	5.9 ± 0.4
	1000 (≒48 μg [10B]/mL)	12.8 ± 1.1
	2000 (≒96 μg [10B]/mL)	23.6 ± 1.7
SAS	500 (≒24 μg [10B]/mL)	10.8 ± 1.8
	1000 (≒48 μg [10B]/mL)	19.1 ± 0.5
	2000 (≒96 μg [10B]/mL)	36.6 ± 1.2

U-87 MG, FaDu, and SAS cells were treated with BPA at 500, 1000, and 2000 μg/mL for 3 h, respectively. Data are represented as the mean ± SD (n = 3). n, number of samples.

**Table 3:** Boron (^10^B) concentration studies in the U-87 MG subcutaneous xenograft model.

BPA Injection (mg/kg)	Time (h)	Boron Concentration (ppm)			T/B Ratio
		Tumor	Blood	Skin	
500	0.17	21.1 ± 4.3	33.3 ± 3.5	26.4 ± 5.9	0.6
	0.5	25.2 ± 12.4	14.4 ± 2.4	20.0 ± 3.1	1.7
	1	23.7 ± 5.1	8.3 ± 0.3	13.9 ± 5.9	2.8
	2	16.4 ± 2.2	7.4 ± 1.3	10.9 ± 6.0	2.2
	3	12.8 ± 3.0	5.5 ± 1.0	7.4 ± 1.0	2.3
	5	10.7 ± 1.5	3.9 ± 0.4	6.6 ± 1.6	2.7
1000	0.17	48.6 ± 11.4	82.1 ± 10.2	52.6 ± 9.5	0.6
	0.5	40.6 ± 9.9	25.2 ± 3.2	29.0 ± 7.0	1.6
	1	40.8 ± 7.8	19.9 ± 4.5	27.5 ± 5.4	2.1
	2	28.5 ± 4.6	13.5 ± 2.8	22.8 ± 3.0	2.1
	3	23.2 ± 3.0	10.9 ± 2.7	17.9 ± 2.3	2.1
	5	8.3 ± 1.9	3.7 ± 1.6	6.2 ± 2.5	2.3

Boron concentration in tumor, blood, and skin after i.v. injection of BPA 500 and 1000 mg/kg and T/B ratio. BPA 500 mg/kg administration group, n = 4; BPA 1000 mg/kg administration group, n = 3–4. Data are represented as the mean ± SD. n, number of animals.

## Data Availability

All the data supporting the conclusions of this article are included within the article.

## References

[1] Mirazaei H.R., Sahebkar A., Salehi R., Nahand J.S., Karimi E., Jaafari M.R., Mirzaei H. (2016). Boron neutron capture therapy: Moving toward targeted cancer therapy. J. Cancer Res. Ther..

[2] Nedunchezhian K., Aswath N., Thiruppathy M., Thirugnanamurthy S. (2016). Boron neutron capture therapy—A literature review. J. Clin. Diagn Res..

[3] Malouff T.D., Seneviratne D.S., Ebner D.K., Stross W.C., Waddle M.R., Trifiletti D.M., Krishnan S. (2021). Boron neutron capture therapy: A review of clinical applications. Front. Oncol..

[4] Miyatake S., Kawabata S., Hiramatsu R., Kuroiwa T., Suzuki M., Kondo N., Ono K. (2016). Boron neutron capture therapy for malignant brain tumors. Neurol. Med. Chir..

[5] Locher G.L. (1936). Biological effects and therapeutical possibilities of neutrons. Am. J. Roentgenol. Radium. Ther..

[6] Sweet W.H. (1951). The uses of nuclear disintegration in the diagnosis and treatment of brain tumor. N. Engl. J. Med..

[7] BNCT History NCBJ. https://www.ncbj.gov.pl/en/bnct/history.

[8] González S.J., Bonomi M.R., Santa Cruz G.A., Blaumann H.R., Calzetta Larrieu O.A., Menéndez P., Jiménez Rebagliati R., Longhino J., Feld D.B., Dagrosa M.A. (2004). First BNCT treatment of a skin melanoma in Argentina: Dosimetric analysis and clinical outcome. Appl. Radiat. Isot..

[9] Kankaanranta L., Seppälä T., Koivunoro H., Saarilahti K., Atula T., Collan J., Salli E., Kortesniemi M., Uusi-Simola J., Välimäki P. (2012). Boron neutron capture therapy in the treatment of locally recurred head-and-neck cancer: Final analysis of a phase I/II trial. Int. J. Radiat. Oncol. Biol. Phys..

[10] Miyatake S., Kawabata S., Hiramatsu R., Furuse M., Kuroiwa T., Suzuki M. (2014). Boron neutron capture therapy with bevacizumab may prolong the survival of recurrent malignant glioma patients: Four cases. Radiat. Oncol..

[11] Hiratsuka J., Kamitani N., Tanaka R., Yoden E., Tokiya R., Suzuki M., Barth R.F., Ono K. (2018). Boron neutron capture therapy for vulvar melanoma and genital extramammary Paget’s disease with curative responses. Cancer Commun..

[12] Wang L., Liu Y.H., Chou F., Jiang S. (2018). Clinical trials for treating recurrent head and neck cancer with boron neutron capture therapy using the Tsing-Hua Open Pool Reactor. Cancer Commun..

[13] Chen Y., Lee Y., Lin C., Pan P., Chen J., Wang C., Hsu S., Kuo Y., Lan T., Hsu S.P.C. (2021). Salvage Boron Neutron Capture Therapy for Malignant Brain Tumor Patients in Compliance with Emergency and Compassionate Use: Evaluation of 34 Cases in Taiwan. Biology.

[14] Suzuki M. (2020). Boron neutron capture therapy (BNCT): A unique role in radiotherapy with a view to entering the accelerator-based BNCT era. Int. J. Clin. Oncol..

[15] Kreiner A.J., Bergueiro J., Cartelli D., Baldo M., Castell W., Asoia J.G., Padulo J., Sandin J.C.S., Igarzabal M., Erhardt J. (2016). Present status of accelerator-based BNCT. Rep. Pract. Oncol. Radiother..

[16] Hirose K., Konno A., Hiratsuka J., Yoshimoto S., Kato T., Ono K., Otsuki N., Hatazawa J., Tanaka H., Takayama K. (2021). Boron neutron capture therapy using cyclontron-based epithermal neutron source and borofalan (10B) for recurrent of locally advanced head and neck cancer (JHN002): An open-label phase II trial. Radiother. Oncol..

[17] Dymova M.A., Taskaev S.Y., Richter V.A., Kuligina E.V. (2020). Boron neutron capture therapy: Current status and future perspectives. Cancer Commun..

[18] Barth R.F., Mi P., Yang W. (2018). Boron delivery agents for neutron capture therapy of cancer. Cancer Commun..

[19] Hoppenz P., Els-Heindl S., Beck-Sickinger A.G. (2020). Peptide-Drug Conjugates and their targets in advanced cancer therapies. Front. Chem..

[20] Takeuchi K., Hattori Y., Kawabata S., Futamura G., Hiramatsu R., Wanibuchi M., Tanaka H., Masunaga S., Ono K., Miyatake S. (2020). Synthesis and evaluation of dodecaboranethiol containing kojic acid (KA-BSH) as a novel agent for boron neutron capture therapy. Cells.

[21] Michiue H., Kitamatsu M., Fukunaga A., Tsuboi N., Fujimura A., Matsushita H., Igawa K., Kasai T., Kondo N., Matsui H. (2021). Self-assembling A6K peptide nanotubes as a mercaptoundecahydrododecapborate (BSH) delivery system for boron neutron capture therapy (BNCT). J. Control. Release.

[22] Häfliger P., Charles R. (2019). The L-type amino acid transporter LAT1-an emerging target in cancer. Int. J. Mol. Sci..

[23] Wongthai P., Hagiwara K., Miyoshi Y., Wiriyasermkul P., Wei L., Ohgaki R., Kato I., Hamase K., Nagamori S., Kanai Y. (2015). Boronophenylalanine, a boron delivery agent for boron neutron capture therapy, is transported by ATB0, +, LAT1 and LAT2. Cancer Sci..

[24] Lee C., Moon M., Lee D.W., Kim H.S., Kwon H., Lee P., Kim D.S., Seo H.J., Hong B.H., Lee H. (2021). Status of development and planning activities on CANS in Korea. J. Neutron. Res..

[25] (2018). Institute for Basic Science (IBS): Overview of the A-BNCT System in Korea. https://indico.ibs.re.kr/event/191/attachments/405/493/WG5-8-AFAD2018-DS_Kim.pdf.

[26] Rogus R.D., Harling O.K., Yanch J.C. (1994). Mixed field dosimetry of epithermal neutron beams for boron neutron capture therapy at the MITR-II research reactor. Med. Phys..

[27] Matsubayashi N., Tanaka H., Takata T., Okazaki K., Sakurai Y., Suzuki M. (2020). Development of real-time neutron detectors with different sensitivities to thermal, epithermal, and fast neutrons in BNCT. Radiat. Meas..

[28] Watanabe K., Kawabata Y., Yamazaki A., Uritani A., Iguchi T., Fukuda K., Yanagida T. (2015). Development of an optical fiber type detector using a Eu:LiCaAlF6 scintillator for neutron monitoring in boron neutron capture therapy. Nucl. Instrum. Methods Phys. Res. A.

[29] Rafehi H., Orlowski C., Georgiadis G.T., Ververis K., El-Osta A., Karagiannis T.C. (2011). Clonogenic assay: Adherent cells. J. Vis. Exp..

[30] Franken N.A.P., Rodermond H.M., Stap J., Haveman J., Bree C.V. (2006). Clonogenic assay of cells in vitro. Nat. Protoc..

[31] Kumada H., Takada K. (2018). Treatment planning system and patient positioning for boron neutron capture therapy. Radiol. Oncol..

[32] Wittig A., Sauerwein A., Coderre J.A. (2000). Mechanisms of Transport of p-Borono-Phenylalanine through the Cell Membrane In Vitro. Radiat. Res..

[33] Yamamoto N., Masunaga S., Kato I., Iwai S., Nakazawa M., Ono K., Yura Y. (2014). Enhancing effect of ultrasound on boron concentrations in an oral squamous cell carcinoma cell line SAS for boron neutron capture therapy. J. Oral. Maxillofac. Surg. Med. Pathol..

[34] Fukuo Y., Hattori Y., Kawabata S., Kashiwagi H., Kanemitsu T., Takeuchi K., Futamura G., Hiramatsu R., Watanabe T., Hu N. (2020). The Therapeutic Effects of Dodecaborate Containing Boronophenylalanine for Boron Neutron Capture Therapy in a Rat Brain Tumor Model. Biology.

[35] Imamichi S., Chen L., Ito T., Tong Y., Onodera T., Sasaki Y., Nakamura S., Mauri P., Sanada Y., Igaki H. (2022). Extracellular Release of HMGB1 as an Early Potential Biomarker for the Therapeutic Response in a Xenograft Model of Boron Neutron Capture Therapy. Biology.

[36] Kanemitsu T., Kawabata S., Fukumura M., Futamura G., Hiramatsu R., Nonoguchi N., Nakagawa F., Takata T., Tanaka H., Suzuki M. (2019). Folate receptor-targeted novel boron compound for boron neutron capture therapy on F98 glioma-bearing rats. Radiat. Environ. Biophys..

[37] Sumitani S., Nagasaki Y. (2012). Boron neutron capture therapy assisted by boron-conjugated nanoparticles. Polym. J..

[38] He H., Li J., Jiang P., Tian S., Wang H., Fan R., Liu J., Yang Y., Liu Z., Wang J. (2021). The basis and advances in clinical application of boron neutron capture therapy. Radiat. Oncol..

[39] Puris E., Gynther M., Auriola S., Huttunen K.M. (2020). L-type amino acid transporter 1 as a target for drug delivery. Pharm. Res..

[40] Barth R.F., Yang W., Rotaru J.H., Moeschberger M.L., Boesel C.P., Soloway A.H., Joel D.D., Nawrocky M.M., Ono K., Goodman J.H. (2000). Boron neutron capture therapy of brain tumors: Enhanced survival and cure following blood-brain barrier disruption and intracarotid injection of sodium borocaptate and boronophenylalanine. Int. J. Radiat. Oncol. Biol. Phys..

[41] Carlsson J., Forssel-Aronsson E., Glimelius B. (2002). The swedish cancer society investigation group, Radiation therapy through activation of stable nuclides. Acta Oncol..

[42] Yoshimoto M., Honda N., Kurihara H., Hiroi K., Nakanura S., Ito M., Shikano N., Itami J., Fujii H. (2018). Non-invasive estimation of 10B-4-borono-L-phenylalanine-derived boron concentration in tumors by PET using 4-borono-2-18F-fluoro-phenylalanine. Cancer Sci..

[43] Blue T., Yanch J.C. (2003). Accelerator-based epithermal neutron sources for boron neutron capture therapy of brain tumors. J. Neurooncol..

[44] Naito F. (2018). Introduction to accelerators for boron neutron capture therapy. Radiol. Oncol..

[45] Maliszewska-Olejniczak K., Kaniowski D., Araszkiewicz M., Tymińska K., Korgul A. (2021). Molecular mechanisms of specific cellular DNA damage response and repair induced by the mixed radiation field during boron neutron capture therapy. Front. Oncol..

[46] Aslam A., Kakakhel M.B., Shahid S.A., Younas L., Zareen S. (2016). Soft tissue and water substitutes for megavoltage photon beams: An EGSnrc-based evaluation. J. Appl. Clin. Med. Phys..

[47] Safavi-Naeini M., Chacon A., Guatelli S., Franklin D.R., Bambery K., Gregoire M., Rosenfeld A. (2018). Opportunistic dose amplification for proton and carbon ion therapy via capture of internally generated thermal neutrons. Sci. Rep..

[48] PMDA Steboronin CTD 2.6: Summary Text and Summary Table of Non-Clinical Studies. https://www.pmda.go.jp/drugs/2020/P20200410001/370738000_5130108021353_H100_1.pdf.

[49] Wang P., Zhen H., Jiang X., Zhang W., Cheng X., Guo G., Mao X., Zhang X. (2010). Boron neutron capture therapy induces apoptosis of glioma cells through Bcl-2/Bax. BMC Cancer.

[50] Lin Y.C., Wang S.J., Chung H.P., Liu H.M., Chou F.I. (2011). Low dose of gamma irradiation enhanced boronophenylalanine uptake in head and neck carcinoma cells for boron neutron capture therapy. Appl. Radiat. Isotopos..

[51] Moro D., Colautti P., Lollo M., Esposito J., Conte V., De Nardo L., Ferretti A., Ceballos C. (2009). BNCT dosimetry performed with a mini twin tissue-equivalent proportional counters (TEPC). Appl. Radiat. Isot..

